# Interface Controlled Micro- and Macro-Mechanical Properties of Vibration Processed Carbon Fiber/Epoxy Composites

**DOI:** 10.3390/polym13162764

**Published:** 2021-08-17

**Authors:** Xiaobo Yang, Lihua Zhan, Yifeng Peng, Cong Liu, Rui Xiong

**Affiliations:** 1 College of Mechanical and Electrical Engineering, Central South University, Changsha 410083, China; lzjt_yangxb@163.com (X.Y.); 193812036@csu.edu.cn (Y.P.); 15842820170@163.com (C.L.); xylx3399@163.com (R.X.); 2 Institute of Light Alloy, Central South University, Changsha 410083, China

**Keywords:** carbon fiber reinforced plastics composite, random vibration assisted vacuum processing, fiber-resin interface, mechanical properties, fiber volume fraction

## Abstract

The fiber-resin interface is an important component that significantly affects mechanical properties of composites. Random vibration-assisted vacuum processing (RVAVP), a new method to improve the adhesion of the fiber-resin interface, was presented. The effects of different curing processes on mechanical properties were comprehensively assessed by combining the fiber push-out test, finite element model simulation, cure monitoring approach, and short-beam three-point bending test, and the correlation between fiber volume fraction and mechanical properties was quantified by a facile thermogravimetric analysis-based methodology. The results revealed that application of random vibration during the curing process can promote the impregnation of resin into fibers and impede the growth of interface defects while improving mechanical properties at the same time. For this reason, the laminates produced by RVAVP exhibited the average interfacial shear strength of 78.02 MPa and the average interface fracture toughness of 51.7 J/m^2^, which is obtained a 48.26% and 90.77% improvement compared with the 0 MPa autoclave process. With the large observed increase in micro-mechanical properties, the average interlaminar shear strength of 93.91 MPa showed a slight reduction of 5.07% compared with the 0.6 MPa autoclave process. Meanwhile, the mechanical properties tended to be stable at the fiber volume fraction of 65.5%.

## 1. Introduction

Carbon fiber-reinforced plastics (CFRP) composites have received considerable attention for the last decade due to a combination of attractive physical-chemical properties such as high-specific strength and modulus, high chemical stability, and corrosion resistance [1]. Because of these superior properties, CFRP composites are considered to be the most important industrial materials for long-term application in the industries of aeronautic and aerospace, as well as other high-tech fields [2,3]. CFRP composites contain a high fiber-volume fraction, which results in a very large interface area per unit of volume [4]. Therefore, the ultimate strength, durability, and multifunctional application of composite parts, to a large extent, are controlled by the interfacial bonding properties [5,6].

A desirable fiber-resin interface formation in the curing process of CFRP composites can be mainly divided into two parts: one is sufficient wetting of fibers by the resin matrix during the impregnation stage, and the other is chemical bonding through the cross-linking reaction of resin during the solidification stage [1,2,3,7,8]. Currently, the quality of the fiber-resin interface in composite structures strongly depends on the temperature and pressure cycle imposed by autoclave during the manufacturing process [7,8]. If the hydrostatic pressure of resin is insufficient due to the autoclave pressure transfer restricted by structural complexity and unreasonable mold design, the infiltration of resin to fibers is inadequate, yielding weak adhesion between the fibers and resin [8,9]. However, if the autoclave pressure is too high, fiber bridge, resin starvation, and other new defects may occur in composite structures during the curing process, which is unfavorable for mechanical properties [10,11,12].

From the development of composite processes of various nature, the increasing of the adhesion between fibers and matrix has been one of the major factors [13,14,15]. The composite community develops alternative out-of-autoclave (OOA) composite manufacturing technologies in order to solve problems brought from the autoclave process [16,17,18]. Vacuum-assisted rein-transfer molding (VARTM) is a method with which resin-transfer power and injection pressure can be adjusted to enhance the wettability between carbon fibers and resin [16,17,19]. In the case of the microwave curing process, the matrix is predominantly heated from fibers and the surrounding interface of fiber is totally cured by a higher temperature and heating rate, resulting in the interfacial bonding between fiber and matrix being improved [20,21]. Note that the OOA processes rely on vacuum bag pressure to form composite structures, while only vacuum bag pressure cannot compact composite structures completely and drives resin fill to gaps between fibers, leading to the growth of micro-defects at the fiber-resin interface during curing process, which results in the improvement of interfacial adhesion being insignificant [22,23,24]. Therefore, a few research institutes attempt to introduce dynamic means into the OOA process, which helps composite structures to obtain a significant decrease of manufacturing defects, a more effective resin impregnation of fibers, and the improvement of interlaminar shear properties with low forming pressure [25,26,27,28]. Despite that, compared with the application of vibration concentrated in the resin-transfer molding process, it is still a challenging way to introduce mechanical vibration to enhance the adhesion between fibers and the resin matrix during the curing process of composite preforms laid by aeronautical prepreg. First, the higher processing temperature of aeronautical epoxy-based prepreg (around 180 °C) further increases the difficulties and expenses of applying vibration in the manufacturing process due to the need for using the high-temperature vibration equipment instead of ordinary equipment during the curing cycle [25]. Second, a better resin flow would facilitate the surface wetting of fibers by resin easily in a low-viscosity medium under vibration [27,28], but the advantage of vibration is not defined during the curing process as a consequence of the low initial flow velocity of the resin matrix within composite preforms fabricated by prepreg. Moreover, although previous researchers developed the relationship between the final measurement of void content and frequency [25,26,27,28], the potential effects that the vibration has on the micro-mechanical properties, micro-structure, and evolution of interface between the fiber and matrix are still rarely performed, especially when they are captured by the novel micro-characterization technologies.

Motivated by these ideas, the purpose of this work was to study the effect of the random vibration on micro and macro mechanical properties of composite laminates produced by RVAVP in the vibration acceleration range of 5–15 g. For the sake of property comparison, identical static samples were produced in this work by an autoclave process with different pressures, including 0 MPa, 0.2 MPa, 0.4 MPa, and 0.6 MPa. Nanoindentation was used to characterize the interfacial shear strength (IFSS), and experimental results were fitted to the finite element model (FEM) to determine the interface fracture toughness. A cure-monitoring approach was used to investigate the in-situ behavior of the evolution of the fiber-resin interface in the different curing processes. Moreover, the interlaminar shear strength (ILSS) of laminates was measured by short-beam three point bending tests coupled with the digital-image correction (DIC) method, and fracture surfaces of tested specimens were characterized by scanning electron microscopy (SEM). Finally, the fiber volume fraction obtained from a thermogravimetric analysis (TGA)-based methodology was correlated to the micro and macro mechanical properties.

## 2. Experimental and Modeling Procedures

### 2.1. Laminate Fabrication Process

The material used in the present study is unidirectional carbon fiber-reinforced thermosetting prepreg (purchased from Commercial Aircraft Co., Ltd., Shanghai, China) consisting of polyacrylonitrile-based T800 carbon fibers of approximately 5 μm in diameter and X850 toughened epoxy resin. The initial fiber volume fraction of prepreg is 65%, and thermo-elastic material properties are presented in details elsewhere [7,8] and are summarized in Table 1. Composite preforms of dimension 200 mm × 200 mm × 2 mm (length × width × thickness) were stacked using lay-ups of [0]_10_ in an ambient environment containing 22 °C and 50% RH.

**Figure 1:**
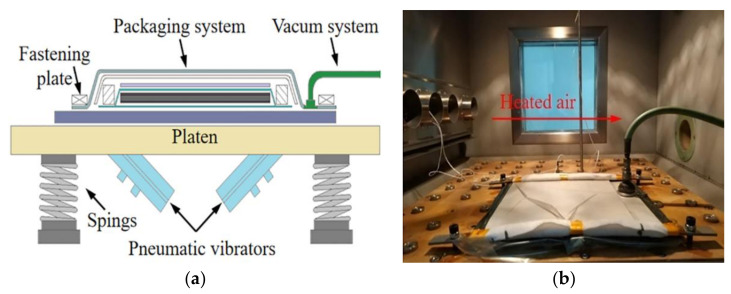
Tooling and preparation for the RVAVP, (**a**) schematic illustration of the experimental-set up configuration, (**b**) a picture of the temperature chamber.

The equipment used for RVAVP was a high-performance repetitive shock (RS) machine designed by the National University of Defense Technology, and the vibration subsystem consisted of a platen with pneumatic vibrators and springs beneath it, as shown in Figure 1a. The random vibration-curing system can provide vibration acceleration from 0 g to 70 g with a frequency range from 10 Hz to 5 KHz and a maximum temperature of 200 °C. During the vibration, the platen was impacted by pneumatic vibrators, and the packaging system was fixed on the platen by fastening plates in order to solve the response distortion of vibration signals. According to the vibration control adopted by the closed-loop feedback strategy, the response acceleration of composite preform calculated by Equation (1) was adjusted to the expected value [29,30]. In the temperature chamber shown in Figure 1b, the electric hot wire was employed to heat the composite preform.

(1) $$G_{rms}={\left[\int_{f_{2}}^{f_{1}}g_{psd}\left(f\right)d\left(f\right)\right]}^{\frac{1}{2}},$$

where, $G_{rms}$ is the root mean square of acceleration (g), $f_{1}$ is lower limit frequency (Hz), $f_{2}$ is upper limit frequency (Hz), $g_{psd}$ is power spectral density (g^2^/Hz).

**Figure 2:**
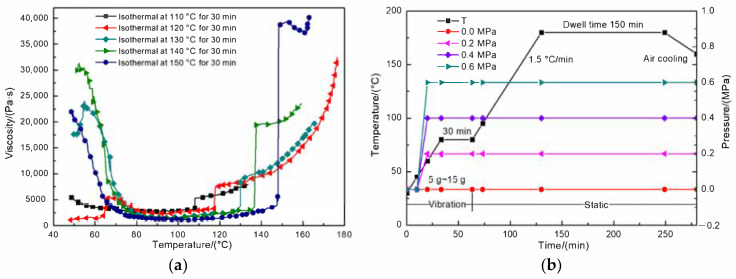
(**a**) Viscosity curves with different curing processing highlighted in Ref [31], (**b**) curing cycles for RVAVP and autoclave process.

The viscosity profiles of the T800/X850 prepreg during simulation of the different cure cycles identified in Ref [31] are shown in Figure 2a. It is evident in Figure 2a that the viscosity begins to reach a plateau that viscosity decreases slowly with the increase of temperature when the temperature reaches 80 °C, and thereafter, the initial stages of dwelling at high temperature for 30 min cause a sudden increase in viscosity. A number of papers, however, have suggested that higher resin viscosity resulting from dwelling at high temperature contributes to incomplete fiber wetting and weak fiber-resin interfacial adhesion [1,7,22,31]. Therefore, the composite laminates were manufactured by a curing process using an adjusted heat treatment cycle with 30 min dwell time at 80 °C, 150 min dwell time at 180 °C, and a heating rate of 1.5 °C/min, as shown in Figure 2b. Random vibrations with three different vibration accelerations, including 5 g, 10 g, and 15 g (g = 9.8 m/s^2^) were chosen to investigate the influence of vibration on the final micro and macro mechanical properties of composite laminates, and the vibration system was run during the curing cycle until 30 min after the core temperature of the preform reached 80 °C. Consolidation pressure was achieved via a vacuum bag during the curing process, which was able to apply approximately 0.1 MPa of external pressure. Besides, when the consolidation pressure that was due to the addition of autoclave pressure and vacuum bag pressure exceeded 0.7 MPa, the effect of pressure on mechanical properties was not significant [7,10,32]. Therefore, identical static samples were produced using autoclave pressures of 0 MPa, 0.2 MPa, 0.4 MPa, and 0.6 MPa in an autoclave following the same temperature cycle, and the vacuum was pulled continuously during all autoclave processes.

### 2.2. Micro-Mechanical Property Testing Method

**Figure 3:**
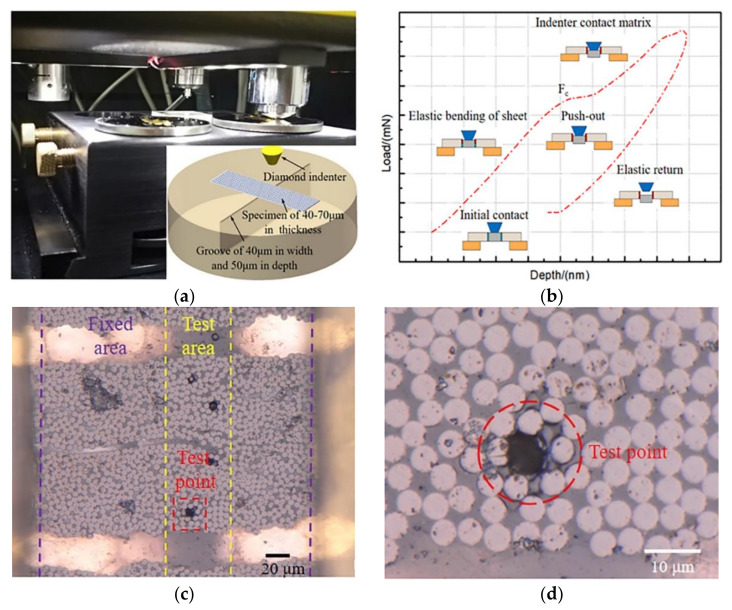
The single fiber push-out test, (**a**) experimental set-up used for the push-out test, (**b**) representative load-displacement curve of the push-out test, (**c**) 1000 times microscopy imaging of the frontside surface, (**d**) 2000 times microscopy imaging showing the detail of the target fiber after the test.

The single fiber push-out tests were performed in the composite cross-sections also in the Nano Indenter XP system, but using a 5 mm flat-end diamond indenter, to quantify the IFSS between the fiber and matrix of composite laminates produced by different curing processes. The test was in load-control mode, with a constant load rate of 1 mN/s up to a maximum load of 120 mN. The selected samples were sliced from composite laminates normal to the fiber direction into 1 mm-thick sections, and then samples were carefully polished to a final thickness of approximately 40–70 μm. The polished samples were mounted on the top of a special platform with a groove of 40 μm in width, as shown in Figure 3a. A number of at least 30 fibers of comparable cross-section area were performed for each composite sample. Figure 3b plots a typical load-displacement curve recorded during a push-out test in loading control. This section of the curve is associated with the elastic of loaded fiber, the debonding of fiber, and deformation of the resin matrix by the action of indenter. Note that the linear region ends with the debonding of the fiber from the matrix and the sliding of fiber. Since push-out tests were performed under a constant loading rate, the plateaus indicate an abrupt increase of displacements, in other words, a complete fracture of the fiber-resin interfaces. The photographs shown in Figure 3c,d were taken after a plateau appeared in the load-displacement curve, respectively. If the load was applied near the boundary of the target fiber during push-out test, the result should be disregarded. The average IFSS can be derived by [4,6]:

(2) $$\tau_{if}=\frac{F_{c}}{2\pi re},$$

where, $\tau_{if}$ is the IFSS (MPa), $F_{c}$ is the load at which fiber starts to slip though the matrix (N), r is the radius of target fiber (m), and e is the thickness of the sample (m).

**Figure 4:**
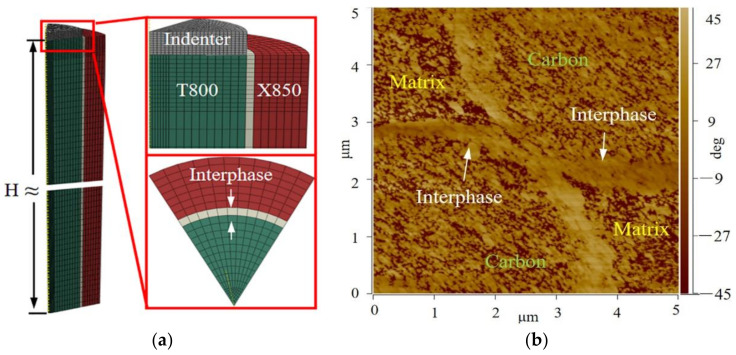
(**a**) Finite-element model for the push-out test, (**b**) atomic force microscopy (AFM) images of composite interface region.

The interface fracture toughness was derived from the obtained load-displacement curve with the help of three-dimensional (3D) numerical simulation of the single-fiber push-out test [33,34,35]. The commercial software package ABAQUS/Explicit V6.14 was used for modeling. The model consists of a flat punch tip and a central fiber embedded in an interfacial layer which is surrounded again by a resin matrix mantle. As shown in Figure 4a, the geometry of the 3D FEM model used to analyze the fiber push-out test was simplified as one sixth of the entire 3D model to improve the computational efficiency by considering the hexagonal symmetry of the fiber. The flat punch tip with a 5 μm diameter is modelled as rigid [33,34,36]. The substructures of the fiber, bond interface zone, and matrix were modeled based on the atomic force microscopy (Figure 4b), which is considered as realistic as possible. Therefore, the fiber had a radius r = 2.5 μm, the thickness of the interfacial layer was 0.08r, and the matrix mantle was 0.56r. Besides, the length parallel to the fiber, H = 16r~28r, was obtained from the actual length of the tested sample. Selected material properties used in the simulations are listed in Table 1. To model the debonding of the fiber from the matrix, the cohesive element layer satisfied with the bilinear traction separation law was attached to the interfacial layer [7,33,34]. The cohesive model relates the total stress acting on the interface, $=\sqrt{\langle t_{n}\rangle {}^{2}+t_{t}^{2}+t_{s}^{2}}$, with the corresponding separation, $\delta =\sqrt{\delta_{n}^{2}+\delta_{t}^{2}+\delta_{s}^{2}}$, where index n refers to the normal direction and t and s are the tangential direction [33,37]. Brackets for $t_{n}$ are the Macaulay brackets, which return the argument if positive and return a zero if negative. Quadratic stress criterion was used to govern the failure initiation, as shown in Equation (3). It is noted that the fiber push-out tests only provided the values of interfacial shear strength. Therefore, isotropic interfacial shear strengths were assumed, specifically $t_{n}^{0}=t_{t}^{0}=t_{s}^{0}=\tau_{if}$ [33,34,37]. Once the damage is initiated, there will be a reduction in the stresses transferred through the interface as well as in the interface stiffness [37,38]. A linear energy criterion was used to capture the softening on the t−δ relation through the interface-fracture toughness value Γ that defines how much energy the interface can absorb before it fails completely [7,33,34,35]. Therefore, the interface fracture toughness of samples produced by different processes can be obtained by calibrating the numerical load-displacement curves with experiment dates [8,35]. The fiber and the matrix were discretized with 8-node linear brick elements (C3D8), except for the interfacial layer, in which 8-node three-dimensional cohesive elements (COH3D8) were used in the interfacial layer. The number of elements in the model was ≈50,000, which is enough to capture the large stress gradients [33]. A ‘hard’ contact condition was defined between the indenter and fiber, and the friction between the indenter and fiber was neglected [33,34]. The interior face was created based on the element set, which is included in general contact [7,8,39]. Additionally, the friction coefficient of element-to-element was set to 0.4 [7]. Prior to mechanical loading, an initial stress state was introduced based on a cooling from the curing temperature of 180 °C to room temperature [7,33,34].

(3) $${\left\{\frac{\langle t_{n}\rangle }{t_{n}^{0}}\right\}}^{2}+{\left\{\frac{t_{s}}{t_{s}^{0}}\right\}}^{2}+{\left\{\frac{t_{st}}{t_{nt}^{0}}\right\}}^{2}=1,$$

where, $t_{n}^{0}$, $t_{s}^{0}$ and $t_{t}^{0}$ are, respectively, normal, shear, tangential, and critical interfacial strengths.

### 2.3. Macro-Mechanical Property Testing Method

The short-beam three-point bending test was performed following the JC/T 773-2010 standard to quantify the ILSS of composite laminates produced by different processes. Five samples were tested for each composite laminate. The samples were machined and polished to 20 mm in length, 10 mm in width, and 2 mm in thickness. The length direction of samples was parallel to fibers. All tests were carried out at ambient temperature, with the crosshead speed of 1 mm/min and roller support span of 14 mm using a CMT5105 universal testing machine. According to the classical relationship, the ILSS of samples was calculated by Equation (4) [7,40]. Additionally, a digital image correction (DIC) technique was employed to obtain the local surface-strain profile to monitor the crack propagation during short-beam three-point bending test. Prior to DIC measurements, a thin layer of white paint was sprayed on the surface using an airbrush gun. The strain evolution of the monitored region was observed by a 1624 × 1236 pixel charge coupled-device camera with a sampling rate of one image per second. The evolution of strain field data was post-processed with the commercially available DIC software (ARAMIS). Besides, the vertical fracture surfaces (Y-Z) and horizontal fracture surfaces (X-Y) of tested samples were characterized by SEM.

(4) $$\tau_{il}=\frac{3F_{p}}{4bh},$$

where, $\tau_{il}$ is the ILSS (MPa), $F_{p}$ is the peak load (N), b is the measured sample width (mm), and h is the measured sample thickness (mm).

### 2.4. Composite Fiber Volume Fraction Measurement

A facile TGA-based methodology highlighted in Refs [28,41] was used to determine the fiber volume fraction of composite laminates. A STA 449C simultaneous thermal analyzer was used for this purpose. More than five samples were tested for each composite laminate. To ensure the maximum heat transfer surface area, samples were machined to a tiny cylindrical shape with weight of around 20 mg, which was large enough to fit a TGA pan. Resin samples were extracted from the prepreg using the methodology proposed in Ref [23,24]. Resin samples and composite samples were separately heated from ambient temperature to 800 °C at a constant heating rate of 10 °C/min under the argon atmosphere with a flow rate of 50 mL/min until the end. When a relative flat region of TGA was observed due to the unchanged mass loss rate during the decomposition of the sample, the inflection point at the selected curve was obtained [41]. The fiber volume fraction could be calculated according to Equation (5) [41].

(5) $$\%W_{fiber\;TGA}=\frac{Residue_{composite}-Residue_{epoxy\;matrix}}{100-Residue_{epoxy\;matrix}}\times 100,$$

where, $\%W_{fiber\;TGA}$ is the amount of the fibers present of composite samples in weight percentage, $Residue_{composite}$ is the residue mass of composite in percentage, and $Residue_{epoxy\;matrix}$ is the residue epoxy matrix in percentage.

## 3. Results and Discussion

### 3.1. Micro Interfacial Shear Property Analysis

#### 3.1.1. Interfacial Shear Strength

**Figure 5:**
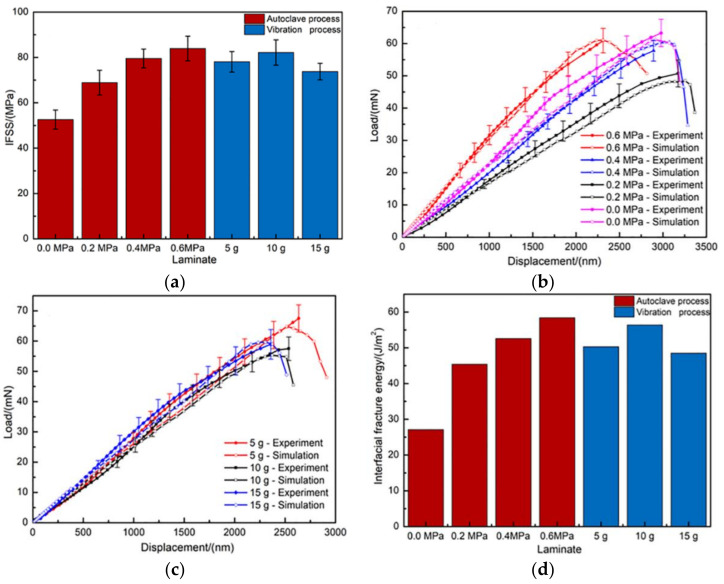
(**a**) IFSS of composites produced by different curing cycles, (**b**) experimental and numerical load-displacement curves of push-out tests for composites produced by the autoclave process, (**c**) experimental and numerical load-displacement curves of push-out tests for composites produced by RVAVP, (**d**) interface fracture toughness of composites produced by different curing cycles.

It is well known that a desirable strength of the fiber-resin interface would be one which is not only critical for ensuring efficient load transfer from the matrix to the fiber, but also sufficiently strong to prevent interface debonding and cracks deflection. Thus, the interface bonding of the composites produced by different processes was quantified through the fiber push-out test in order to relate the interfacial shear property to other mechanical properties, especially the interlaminar shear strength of composites. According to Equation (2), the IFSS of composites as a function of different processes was determined, and is plotted in Figure 5a. The interfaces in the composites produced by the 0 MPa autoclave process were quite weak, having an IFSS of 52.62 MPa. The interface bonding between the fiber and matrix increases with the increase of the autoclave pressure. When the autoclave pressure increased to 0.6 MPa, the IFSS increased from 52.62 MPa to 83.95 MPa and the IFSS changed by up to 59.54%. At the higher autoclave pressure, resin more easily flows towards fibers and cover fiber surfaces, which improves the adhesion between fibers and the resin matrix during the curing process. When the random vibration was introduced into the curing cycle, the IFSS of composites was remarkably improved and the average growth range of IFSS was 48.26% compared to 0 MPa autoclave pressure. As the vibration acceleration increased from 5 g to 10 g, composites exhibited a very strong interface and the IFSS reached a relatively high value of 82.18 MPa, which showed a slight reduction of 2.15% compared with the 0.6 MPa autoclave process. The sudden enhancement of IFSS can be attributed to the improved adhesion between the fiber and matrix in composites. Applying vibration improves the fluidity of the resin matrix, which enables the fiber areas to be infiltrated by the resin of periodic motion caused by vibration under the vacuum bag pressure during the initial curing stage. As a consequence of the adequate infiltration of resin to fibers, the formation of strong interface bonding between fibers and the matrix increases the bearing capacity in the direction perpendicular to fibers. However, when acceleration increased from 10 g to 15 g, the IFSS decreased from 82.18 MPa to 73.79 MPa with a reduction of 10.21%. This is reasonable considering that good wetting of fibers by the resin matrix during the impregnation stage is a prerequisite to proper interface adhesion [2], while the excess fluidity of the resin matrix caused by 15 g RVAVP may be harmful to the balance of the reversible adhesion energy between fibers and the resin matrix and result in an increase of the number of defects in the interface region during the wetting process.

In order to determine the interface fracture toughness of the composite produced by different processes accurately from experimental load-displacement curves, detailed FEM simulations were performed using the interfacial shear strengths determined by nanoindentation. When the load reaches the critical value ($F_{c}$), which is defined as the threshold between the linear and flat region, the fiber begins to debond off of the resin matrix and fiber-resin bonding is completely lost. For this part, $0\;\leq \;F\;\leq F_{c}$ obtained during the push-out tests for each laminate was used to compare with the simulated curve, as shown in Figure 5b,c. The simulated load-displacement curves showed a sharp load drop at the peak force, $F_{c-FEM}$, due to the total debonding of the interface in FEM models. The simulated load-displacement curves were matched to the experimental one by an iterative procedure in which the interface fracture toughness was varied. Although it was still observed that there was a slight uncertainty in the difference between the experimental data and simulation data, the varied trend of simulation curves was in accordance with the experimental load-displacement curves obtained from experiments. Additionally, the uncertainty of difference can be attributed to the varying surrounding environment of the analyzed fibers, the deviation of nanoindentation site from the center of the tested fiber, and/or the deviation between the local experimental environments and simplified models. The main purpose of the interface simulation was to obtain the interface fracture toughness for fiber-resin interface, and the relationship between different curing processes and interface fracture toughness of composites is plotted in Figure 5d. A minimum value of 27.1 J/m^2^ was obtained for the composite produced by the 0.0 MPa autoclave process, while the formation of the fiber-resin interface by the high-pressure treatment led to a marked increase of interface fracture toughness to 58.4 J/m^2^ for the composite produced by the 0.6 MPa autoclave process. When composite laminates were produced under vibrations at different acceleration with vacuum bag pressure, interface fracture toughness reached the maximum value of 56.4 J/m^2^ at 10 g. Comparable values for a carbon fiber/epoxy-resin interface are reported in the literature with respect to the interface fracture toughness introduced by Greisel et al. [42]. These values are in the range of 24 ± 15 J/m^2^ to 57 ± 23 J/m^2^. The order of the magnitude of the interface fracture toughness values evaluated in the present study is verified by these findings.

**Figure 6:**
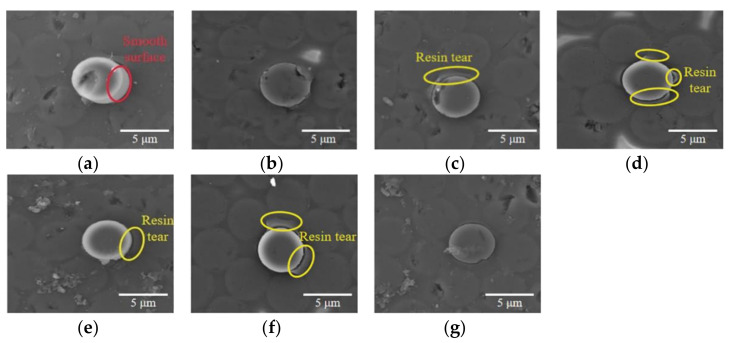
SEM micrographs of the typical pushed fiber, (**a**) 0.0 MPa autoclave process, (**b**) 0.2 MPa autoclave process, (**c**) 0.4 MPa autoclave process, (**d**) 0.6 MPa autoclave process, (**e**) 5 g RVAVP, (**f**) 10 g RVAVP, (**g**) 15 g RVAVP.

Figure 6 shows the debonding behaviors between the carbon fibers and the resin matrix in composites produced by different curing cycles after the push-out tests. On the back side of the sample produced by the 0 MPa autoclave process, the typical pushed-out fiber showed a clean surface, and no evidence of matrix damage was observed around the pushed fiber, as shown in Figure 6a. After the crack ran through the whole interface during the push-out test, the fiber was protruded easily from the surface due to the weak fiber-resin bonding. On the contrary, with the increasing of curing pressure, the pushed-out portion presented partly small debris of resin attached to the separated surface of fibers and the phenomenon of matrix tearing, which indicated that the interfacial debonding failure process of samples produced by high curing pressures needs higher overall energy absorbed as a consequence of the improvement of adhesion between fibers and the matrix during the push-out tests [7,42]. As shown in Figure 6e,f, when a random vibration of no more than 10 g was introduced into the curing cycle, the phenomenon of matrix tearing was observed around the pushed fiber due to the strong adhesion between fibers and the matrix, which is similar to the patterns cured under high autoclave pressures within the range of 0.4 MPa to 0.6 MPa. Once the applied random vibration reached 15 g, the phenomenon of matrix tearing disappeared, which indicated that the weak adhesion between the fiber and resin matrix causes the quick loss of effectiveness of the fiber-matrix system during the push-out test.

#### 3.1.2. Composite Microstructure

**Figure 7:**
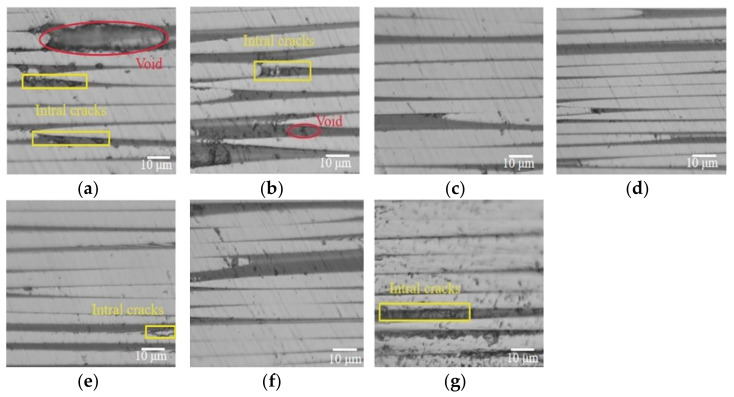
The cross-sectional optical digital micrographs of composite laminates, (**a**) 0.0 MPa autoclave process, (**b**) 0.2 MPa autoclave process, (**c**) 0.4 MPa autoclave process, (**d**) 0.6 MPa autoclave process, (**e**) 5 g RVAVP, (**f**) 10 g RVAVP, (**g**) 15 g RVAVP.

The results discussed above indicated that the RVAVP has a significant improvement on the mechanical property of fiber-resin interface under lower curing pressure compared with the autoclave process, while the IFSS of samples produced by RVAVP showed a nonlinear behavior and reached a maximum value at 10 g. In order to clarify the effect of microstructure of composites produced by different curing cycles on the mechanical property of the fiber-resin interface, the representative cross-sectional morphologies of composite laminates were captured parallel to fibers using an optical digital microscope (model: VHX-5000). As shown in Figure 7a,b, the voids elongated along the fiber direction were distributed in the fiber bundles and micro-cracks appeared between the fiber and the matrix due to insufficient impregnation induced because of the low hydrostatic pressures of resin during the autoclave process. When the fiber bears the axial load during the push-out test, cracks easily propagate from stress concentrations induced by voids, and the existing micro-cracks may become the path of new crack growth [7,35]. As shown in Figure 7c,d, when autoclave pressure reached the range of 0.4–0.6 MPa, the micro-defects around the fiber-resin interface significantly reduced with the increase of pressures. Figure 7e,f show that applying the random vibration into the curing process of composite laminates production improved the resin fluidity, which helps to eliminate micro-defects around the fiber-resin interface and then provides composites with strong adhesion between fibers and the matrix. In these cases, voids and existing micro-cracks are no longer the main reason for push-out failure, and the emanation and propagation of cracks need higher overall energy absorbed during the process of interface debonding and interface sliding [43,44,45]. However, if vibration acceleration exceeded 10 g, the location and characteristics of micro-defects were similar to the pattern cured under low pressures, and these critical imperfections caused by poor wettability of the resin matrix to carbon fibers will induce the fiber-resin interface failure in an easy way.

#### 3.1.3. Fiber-Resin Interface Development

The evolution of the fiber-resin interface within composites is a time-dependent process during the curing cycles, and the in-situ behavior of evolution of the fiber-resin interface is difficult to detect and track in real-time. Therefore, a cure monitoring approach highlighted in Refs. [22,24] was used to investigate the differences of the evolution of the fiber-resin interface between autoclave process-cured samples and RVAVP-cured samples. For this purpose, the curing process of composite laminates was labelled L_1_: 80 °C-0 min, L_2_: 80 °C-15 min, L_3_: 80 °C-30 min, and L_4_: 180 °C-0 min. When the setting point was reached, the curing process was halted, and then laminate was taken out and quenched to approximately 0 °C using iced water. After quenching, the SEM was employed to obtain the in-situ behavior of the fiber-resin interface, which was frozen at a pre-defined point.

**Figure 8:**
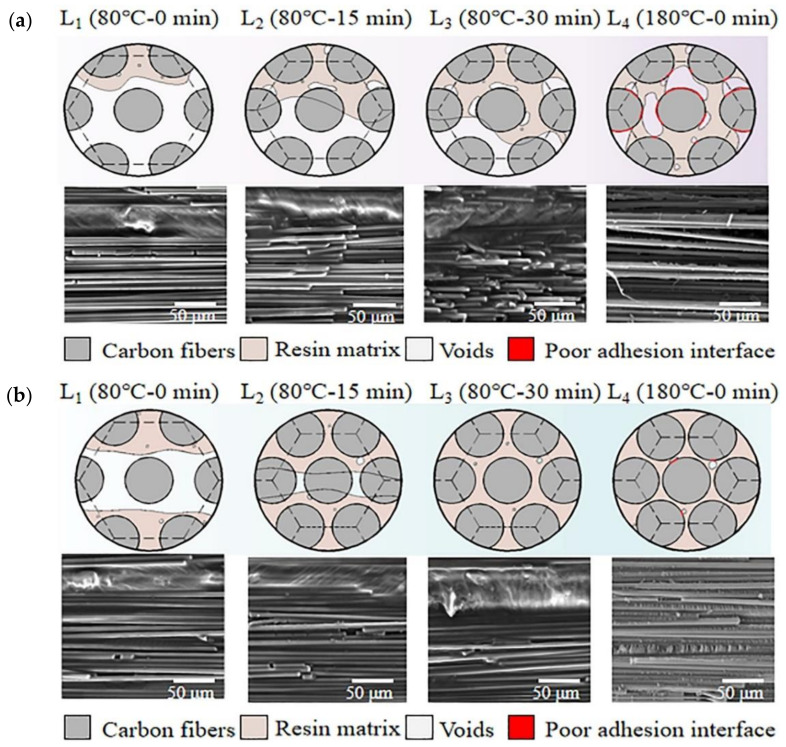
Evolution of the fiber-resin interface within composites during the curing cycles, (**a**) 0.0 MPa autoclave process, (**b**) 10 g RVAVP.

When the hydrostatic pressure of resin was only influenced by the vacuum bag pressure during the curing cycle, the resin flowed from normal to composite laminate and the compaction behavior of the fiber network is shown in Figure 8a. According to the model of one-dimensional consolidation of a composite with three-dimensional flow proposed by Gutowski in Refs. [46,47], the low pressure provides laminate with insufficient fluidity of the resin matrix, causing resin difficulty in infiltrating the fiber bundles and wetting the surface of fibers during the first isothermal dwell (L_1_–L_3_). Meanwhile, it is also difficult for the trapped volatiles in the fiber bundles to be discharged from the vacuum system prior to cure. When the temperature rises from 80 °C to 180 °C (L_3_–L_4_), the sudden increase in resin viscosity influenced by the cross-linking reaction will further suppress the resin infiltration of fibers and the elimination of micro-defects [24]. The lack of external pressure acted on resin leads to insufficient impregnation and allows voids and micro-cracks to remain around the fiber-resin interface, which poses challenges in producing composites with strong adhesion of the fiber-resin interface during the autoclave process with low pressures.

Figure 8b shows the evolution behavior of the fiber-resin interface within composites determined by the cure-monitoring procedure for RVAVP. Applying random vibration into the curing cycle showed an obvious change in the flow of the resin through fiber bundles and an increase of compaction behavior of fibers, which indicated that random vibration contributes towards the increase of fluidity of the resin matrix and, as a result, effective resin impregnation through the fibers was obtained. When the constant pressure changed to a dynamic one, the positive and negative pressure part of vibration enabled the resin to flow towards fiber bundles in the bidirectional way instead of a unidirectional flow. In this case, considering the resin impregnation stage (L_1_–L_3_), it is obvious that fibers were wetted by the resin matrix, and then the fiber surface was coated evenly by a thin layer of the resin matrix in this process. In addition, with the improvement of the ability of the resin to flow through the fiber bundles, resin can be filled in the region where micro-defects occur easily under low pressures, which leads to void traps being very difficult to grow as well as micro-crack inhibition. As the cure-monitoring process reaches the last pre-defined point (L_4_), the sufficient impregnation of the resin matrix into carbon fibers helps develop a better interface adhesion between the fiber and the matrix during the curing reaction [1,24,48].

### 3.2. Macro Interfacial Shear Property Analysis

#### 3.2.1. Interlaminar Shear Strength

**Figure 9:**
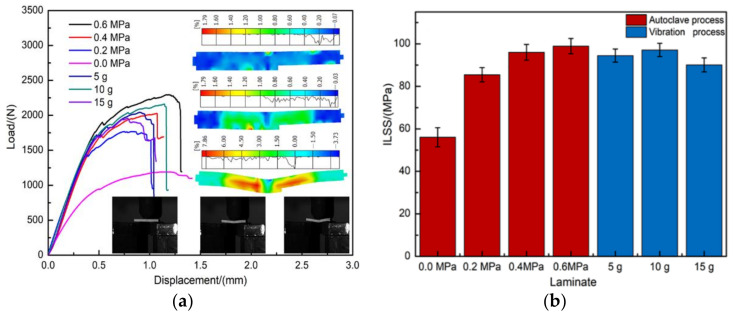
(**a**) Experimental load-displacement curve of composite specimens with different curing cycles; the insert is the result obtained by DIC, (**b**) measurement of ILSS for different curing processes.

The interlaminar shear strength has long been recognized to be crucial for the macro-mechanical limitation of interlaminar fractures related to shearing [7,8,9,40]. In order to study the effect of different curing processes on the interlaminar shear property of these composites, short-beam three-point bending tests were performed on the composite samples. Typical load-displacement curves for specimens during the short-beam three-point bending tests are shown in Figure 9a. The profile of the curves for the composite specimens was approximately similar, in that they all presented initial linear region until the load reached the nonlinear stage, which was immediately followed by a sudden and irreversible load drop. According to the accumulated strain contours obtained by DIC (insert in Figure 9a) during the test, the increase in the strain perpendicular to the loading direction promoted crack initiation between the middle plies, and then the transverse penetration of interlaminar damage at the edge of composite was observed after the shear load reached a certain limit. During short-beam three-point bending tests, the typical pseudo-plastic fracture behavior of composites was in agreement with the results of other test campaigns in Refs. [49,50]. Using Equation (4), the corresponding measured results of ILSS with different curing processes are reported in Figure 9b. It can be clearly seen that the ILSS of composites reached a minimum value of 56.06 MPa at the 0 MPa autoclave process. Additionally, the ILSS of composites increased as autoclave pressure increased and reached a peak value of 98.93 MPa when the autoclave pressure was 0.6 MPa, having a 76.47% improvement compared with the one cured by the 0 MPa autoclave process. For the composites produced by RVAVP, the measured values indicated a standard enhanced interlaminar shear behavior. In comparison with the 0 MPa autoclave process, the composites produced by RVAVP showed, as expected, a stronger response during tests, with an average growth range of 67.51%, and reached the maximum of 97.12 MPa at 10 g. This improvement can contribute to the fact that the random vibration promotes the formation of strong interfacial bonding and eliminates manufacturing defects within composites in comparison with the composites prepared with low pressures during the same time, improving the extrinsic energy absorbed in terms of the cracks expanding and then providing an increase in the ILSS.

#### 3.2.2. Rupture Surface of Composites

**Figure 10:**
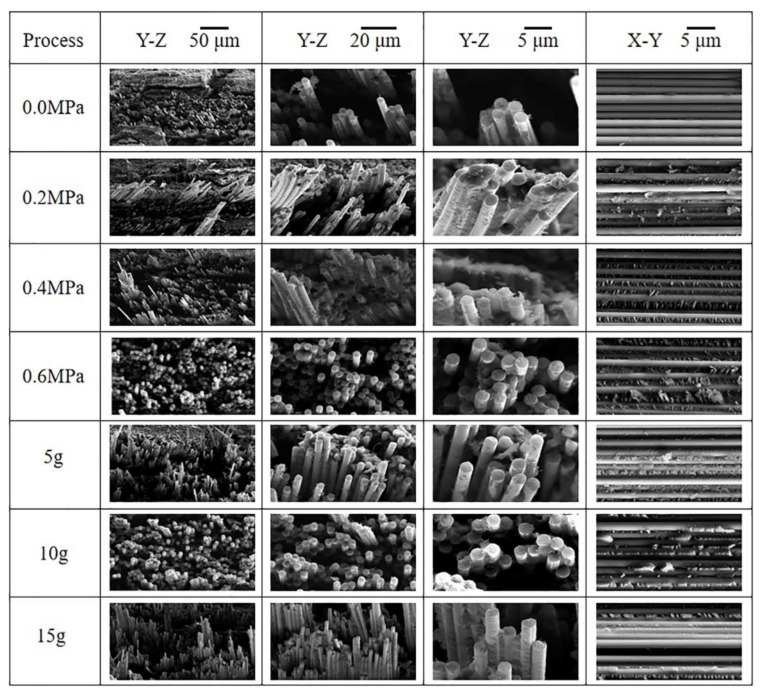
Representative scanning electron micrographs of vertical and horizontal fracture surfaces of composites produced by different curing processes.

In order to better understand the interfacial conditions, SEM images of fracture surfaces of composites including the as-received, autoclave process-cured samples and RVAVP-cured samples, after short-beam three-point bending tests, are shown in Figure 10. The vertical fracture surfaces of composites produced by low autoclave pressures displayed interface debonding and long pull-out fibers, which can be attributed to the weak interfacial bonding in that the interface failed once the cracks emanated and grew to the interface. The horizontal sections showed that some large gaps existed within the fiber bundles, and these gaps mainly originated from the development of the trapped volatiles occurring during the heat-up process and the incomplete infiltration of the resin solution. On the contrary, with the increase of the autoclave pressure, the gradual decrease of fiber pull-out length was observed, which results from the enhanced adhesion between the fibers and resin. When the autoclave pressure reached up to 0.4 and 0.6 MPa, no gaps remained between the fibers, and the fiber surface was adhered by a thick layer of resin matrix. Application of random vibration during the curing process of composites plays a key role in impeding crack energy dissipation mechanisms, such as interface debonding and sliding, fiber pull-out, and endowing composite laminates with excellent interlaminar shear properties. The vertical fracture surface micrographs obtained for the composite produced by RVAVP exhibited the limited pull-out fibers and fracture surfaces looked flat, supporting the relative strong interfacial bonding between fibers and the matrix. Meanwhile, it is very clear from the horizontal fracture surfaces that the successive cusps formed along the main crack propagation direction appeared on the fiber’s surface, which indicates that the exterior load can be transferred efficiently from the matrix to fibers though the fiber-resin interface. Thus, the excellent mechanical properties of reinforcement are utilized, which is responsible for the enhanced carrying capacity of composites. However, a further increase of vibration acceleration leads to ragged fracture surfaces, pull-out fibers, and the decrease of the phenomenon of the successive cusps sticking to the fiber. These observed results are accordance with the weak interfacial shear property of composites [7,8,9].

### 3.3. Correlation of Micro- and Macro-Mechanics

**Figure 11:**
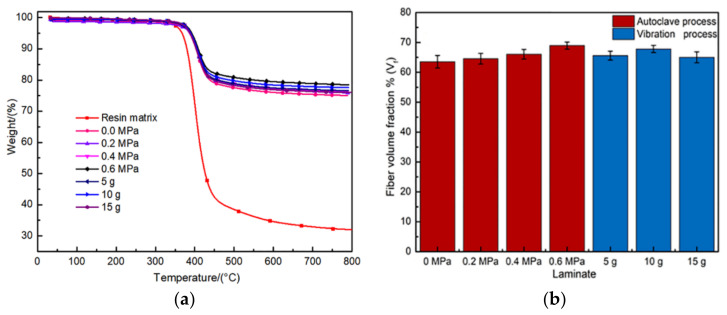
(**a**) The typical TGA response of resin and composite samples, (**b**) fiber volume fraction of the composite samples.

In general, given a specific composite structure, the macro-mechanical property of composite not only depends on the interfacial responses between fibers and the matrix [51], but also includes the fiber volume fraction [52]. During the formation process of the strong fiber-resin interface within composites, the sufficient impregnation of the resin matrix into fiber bundles is accompanied frequently by the phenomenon of multiple fiber-fiber aggregation, which leads to the compaction of fiber layers and an increase of fiber volume fraction [9,46,47]. Therefore, the TGA measurements at the given temperature range from the ambient temperature to 800 °C were carried out to gain more insight into different curing processes-induced effects on the fiber volume fraction. Figure 11a shows the typical mass loss rate versus temperature curves of the resin matrix and composite samples, and all tested samples showed two loss steps. During the first step, samples first showed mass loss most probably due to the moisture elimination [28,41], which was evidenced by the character of slow descent shown by the curves in the earliness. The second one that occurred at around 400 °C was caused by the complete decomposition of resin in a single-step process, and then the curves turned into the flat region can be attributed to a negligible effect of the given temperature range on the mass loss of carbon fibers [41]. After the mass loss rate became stable, the residual weight was used to calculate the fiber weight fraction by Equation (5). As seen in Figure 11b, the fiber volume fraction obtained the minimum value of 63.50% under 0 MPa autoclave pressure. With the increasing of autoclave pressure, the high pressure difference existed along the thickness direction leads that fibers closed more inside composite laminates, which resulted in the peak value of 68.93% at 0.6 MPa. Compared with the autoclave process, the oscillating pressure fields caused by the application of vibration improved the compaction of composites during the curing process. Thereby, the fiber volume fraction values of composites produced by RVAVP scattered more or less around the value of 66.14%, while increasing to 67.79% for the composites produced at 10 g.

**Figure 12:**
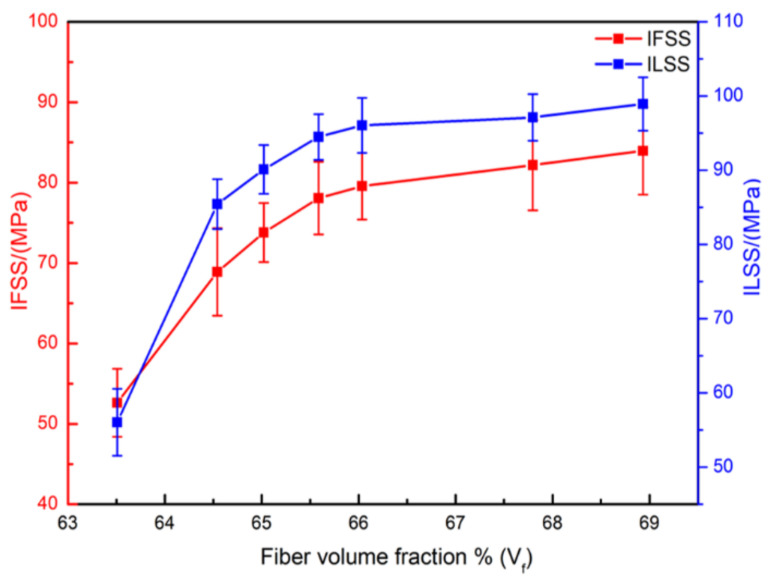
Changes in the IFSS and ILSS of the composite specimens as fiber volume fraction.

The IFSS and ILSS of composites varying with fiber volume fraction are shown in Figure 12. It can be seen that both IFSS and ILSS of composites increased as the fiber volume fraction increased, and the ILSS trend of these composites coincided with the IFSS trend. The enhanced flow-compaction behavior indicates the increase in interface area per unit volume was accompanied by the enhanced interfacial adhesion, which resulted in an improved macro mechanical performance that favors better load transfer between the reinforcements and matrix [33,46]. From this perspective, regardless of whether they were static or vibrated, the composites with excellent mechanical properties showed the propensity to have the high fiber volume fraction. Parallel to this, the micro and macro-mechanical properties tended to be stable when the fiber volume fraction reached 65.5%, which could be recommended as the critical reference for fabricating the high-quality CFRP composites.

## 4. Conclusions

In this present work, the RVAVP was adopted to manufacture the CFRP composite laminates, and identical static samples produced by autoclave processes with different pressures were used for property comparison. The effect of the application of random vibration during the curing process on the interfacial shear properties, composite microstructure, evolution of the fiber-resin interface, macro interlaminar shear property, and fiber volume fraction was studied comprehensively by a novel nano-mechanical testing method (fiber push-out) coupled with the finite-element method, optical digital microscope, cure-monitoring approach, short-beam three-point bending test, thermogravimetric analysis, and etc. The following conclusions can be made based on the obtained results:

(1) Applying random vibration into the curing process of composite laminates production can improve the impregnation of the resin matrix into fiber bundles and impede the growth of micro-defects around the fiber-resin interface, which helps to form a better interface adhesion between the fiber and matrix under low curing pressures. Compared with the 0 MPa autoclave process, the IFSS increased from 52.62 MPa to higher than 78.02 MPa, together with an increase of the interface fracture toughness from 27.1 J/m^2^ to higher than 51.7 J/m^2^.
(2) Due to the strong fiber-resin interfacial interactions, the introduction of random vibration significantly enhanced the composite ILSS to higher than 93.91 MPa, which obtained a slight reduction of 5.07% compared with the one cured by the 0.6 MPa autoclave process. Meanwhile, the flat fracture surfaces with the limited pull-out fibers and significant successive cusps indicated increases in the capability to transfer loads from the matrix to reinforcements through interfaces.
(3) Regardless of whether they were static or vibrated, the sufficient impregnation of the resin matrix into fiber bundles was accompanied by the enhanced flow-compaction behavior during the formation process of the fiber-resin interface. The micro and macro-mechanical properties showed a positive correlation to the fiber volume fraction and tended to be stable when the fiber volume fraction reached 65.5%

## Figures and Tables

**Table 1:** Thermo-elastic constants of the matrix, fibers, and the homogenized composite.

Material	E_1_ (GPa)	E_2_ (GPa)	G_12_ (GPa)	G_23_ (GPa)	v_12_	α_1_ (10^−6^/°C)	α_2_ (10^−6^/°C)
Fiber	282	11.38	6.37	3.84	0.33	−0.56	5.6
Matrix	4.68	4.68	1.80	1.80	0.35	40	40
Interface	10.8	10.8	4.6	4.6	0.18	28	28
Composite	185	9.03	4.75	3.15	0.34	13.6	17.6

## Data Availability

The data presented in this study are available on request from the corresponding author.
